# Impact of a Novel Dietary Supplement on Efficacy of Pharmacological Treatments for Androgenic Alopecia: A Real‐Life, Multicenter, Randomized, Assessor‐Blinded Trial on 225 Subjects

**DOI:** 10.1111/jocd.70388

**Published:** 2025-08-25

**Authors:** Massimo Milani, Stefano Alfano, The AGA‐P Real‐Life Study Group, Laura Alessi, Lisa Cecchini, Annalisa Colantonio, Giulio Cortonesi, Antonio Del Sorbo, Pietro Forleo, Giuseppe Garbo, Valentina Garelli, Sabrina Longhitano, Laura Marchese, Marco Marconi, Fabrizio Presta, Ersilia Tolino

**Affiliations:** ^1^ Medical Department Cantabria Labs Difa Cooper Milano Italy

**Keywords:** androgenic alopecia, controlled trial, *Cucurbita pepo* extract, *Serenoa repens* extract

## Abstract

**Background:**

Minoxidil and finasteride are currently the only FDA‐approved pharmacological treatments for androgenic alopecia (AGA) and female androgenic alopecia (FAGA). However, substantial improvement is observed in no more than 20% of patients in the medium term. To enhance clinical responses, nonpharmacological dietary supplementation is often utilized.

**Aims:**

This study evaluated the efficacy of a novel dietary supplement, AGA‐P, which contains *Serenoa repens* extract, *Cucurbita pepo* extract, L‐Cystine, and Vitamin C, in a multicenter, randomized, assessor‐blinded, real‐life trial alongside pharmacological treatments. The objective was to determine whether dietary supplementation could improve the clinical efficacy of minoxidil and finasteride.

**Patients and Methods:**

A total of 225 subjects with AGA or FAGA (165 men, mean age 40 ± 14 years, range 18–74) were enrolled after obtaining informed consent. Inclusion criteria included male subjects over 18 years and postmenopausal women with mild to moderate AGA/FAGA, eligible for dietary and/or pharmacological treatment. Of these participants, 106 (24 women and 82 men) were assigned to receive pharmacological treatment plus dietary supplementation (one capsule daily; Group A), while 119 (36 women and 83 men) received drug treatment only (Group B). The pharmacological treatments consisted of topical minoxidil and oral finasteride in most cases. Treatment duration was 6 months.

**Results:**

The results indicated that oral supplementation significantly increased the clinical efficacy of pharmacological treatments for mild‐to‐severe AGA/FAGA compared to drug treatment alone (great improvement: Group A 36.5% vs. Group B 25%; *p* = 0.04).

**Conclusion:**

Oral supplementation of AGA‐P significantly increases the clinical efficacy of pharmacological treatments for mild‐to‐severe AGA/FAGA (Study Registration: ISRCTN‐19671217).

## 1 Introduction

Androgenic alopecia (AGA) affects a significant portion of the population, leading to psychological distress and social stigma [1]. Currently, minoxidil and finasteride are the only FDA‐approved treatments for AGA and female androgenic alopecia (FAGA) [2]. However, their long‐term success rates often fall short of patient satisfaction, with only about 20% of patients experiencing substantial improvements in hair density [3]. Emerging research suggests that nonpharmacological interventions, such as dietary supplements, may offer additional benefits for those undergoing pharmacological treatment [4]. This study investigates a novel dietary supplement composed of *Serenoa repens* extract, *Cucurbita pepo* extract, L‐Cystine, and Vitamin C, aimed at enhancing the pharmacological efficacy of existing AGA treatments. *S. repens* acts as a natural 5‐alpha reductase inhibitor, reducing DHT levels and mitigating hair loss [5, 6]. *C. pepo* (pumpkin seed extract) is rich in phytosterols, which are hypothesized to counteract the effects of DHT on hair follicles [7]. L‐Cystine, a sulfur‐containing amino acid, is crucial for keratin synthesis, essential for strong and healthy hair [8]. An increase in cysteine levels can strengthen hair structure and improve overall hair quality [9]. Consequently, the composition of this dietary supplement could represent an interesting therapeutic strategy as an adjuvant treatment for subjects with AGA who are receiving antialopecia medications.

## 2 Objectives

This trial aims to evaluate the impact of the novel dietary supplement (AGA‐P) on the efficacy of pharmacological treatments among subjects diagnosed with mild to severe AGA or FAGA. We hypothesize that the adjunctive use of AGA‐P will raise the rate of clinical improvement as assessed by a seven‐point Global Assessment Scale (GAS).

## 3 Materials and Methods

### 3.1 Study Design

This study was a multicenter, randomized, assessor‐blinded 6‐month trial conducted across outpatients dermatological hair clinics in Italy. The study was conducted between March 2024 and April 2025 in 13 dermatology clinics. Ethical approval was obtained in February 2024 (Trial Registration ISRCTN‐19671217). A total of 225 subjects were enrolled, comprising 60 women and 165 men, with a mean age of 40 ± 14 years, after their written informed consent. The trial was conducted according to the Declaration of Helsinki and Good Clinical Operation procedures [10, 11].

### 3.2 Participants

Participants were included based on the following criteria: Male subjects aged over 18 years or age‐appropriate women diagnosed with AGA/FAGA mild to severe (Hamilton‐Nordwood Classification II–VII; Ludwig Classification: II1‐Advanced) stages of alopecia, appropriate for pharmacological treatment and/or dietary supplementation.

### 3.3 Randomization and Allocation

Participants were randomly assigned (1:1 ratio) to one of two groups: Group A: 106 subjects (24 women and 82 men) received pharmacological treatments along with one capsule of the dietary supplement AGA‐P daily. Group B: 119 subjects (36 women and 83 men) received pharmacological treatment without the supplement. The capsule composition included *S. repens* extract (320 mg), *C. pepo* extract (320 mg), L‐Cystine (425 mg), Vitamin C, and Zinc. The randomization list was generated using a dedicated computer program. Table 1 reports baseline demographic and clinical characteristics of the study population, which were balanced across both groups. Topical minoxidil (5%–2% lotions) was the most used pharmacological treatment (49% in Group A and 52% in Group B).

**TABLE 1:** Demographic and clinical characteristics at baseline.

	Group A (drug treatment and dietary supplementation)	Group B (drug treatment only)
Men/women	82/24	83/36
Mean age	39 ± 14	41 ± 14
*Alopecia severity grade*		
Men (*n*/%) (Norwood‐Hamilton Classification)		
I	2 (2)	3 (4)
II	12 (15)	14 (18)
III	28 (35)	27 (33)
IIIVertex	15 (18)	15 (17)
IV	10 (12)	10 (11)
V	9 (11)	9 (11)
VI	5 (6)	5 (6)
VII	1 (1)	0 (0)
Women (*n*/%) (Ludwig‐Savin Classification)		
II–1	5	9
II–2	10	13
III	9	14
Type of Pharmacological treatments		
Minoxidil (topical)	49%	52%
Finasteride (oral)	5%	6%
Latanoprost	2%	3%
Other galenic formulations	44%	39%

### 3.4 Treatment Duration

Each participant was treated over a period of 6 months. Adherence to the treatment regimen was monitored through regular follow‐ups.

### 3.5 Primary Endpoint

The main endpoint was the percentage of participants achieving a GAS of +3 by month six. GAS was assessed using a seven‐point scale: Much Improved (+3); Moderately Improved (+2); Slightly Improved (+1); Stable (0); Slightly Worsened (−1); Moderately Worsened (−2); Much Worsened (−3). The GAS score was evaluated and graded based on high‐definition color pictures (taken at baseline and at Month 6) of the subjects' scalp (vertex view) by an investigator unaware of treatment allocation group. All photographs were taken in a controlled environment by a dermatologist with a specific background in hair loss research using consistent lighting conditions to minimize shadows. A fixed camera distance of 1 m was maintained at a resolution of 12 megapixels, with aperture and shutter speed settings calibrated uniformly across sessions. Prior to imaging, the scalp was prepared by parting and clipping hair to enhance visibility of affected areas. This standardized approach aims to improve comparability and reliability of trial findings.

### 3.6 Secondary Endpoints

Safety and tolerability were also assessed through side effects and patient‐reported outcomes following the supplementation and drug treatments.

### 3.7 Statistical Analysis and Sample Size Calculations

Statistical analysis was performed using Graph‐Pad statistical software (USA). The primary outcome measure was the percentage of participants achieving a score of +3 points improvement in alopecia severity score (on a seven‐point scale). We hypothesized that the active treatment group would exhibit a greater percentage of participants with significant improvement compared to the control group. According to literature data, we assumed that the control group would have a 20% response rate [12]. To compare the response rates between the two groups, we planned to use a Chi‐square test for categorical outcomes. A two‐sided significance level of 0.05 was considered statistically significant. According to Olsen et al. (2007) minoxidil‐treated subjects obtained a score of +3 in 20% of the cases. Given an expected 20% improvement in the control group and the hypothesis of a 35% improvement in the active group, a total sample size of 210 (105 per groups) was calculated to achieve 80% power to detect a difference in response rates, considering a 1:1 allocation ratio. The sample size calculation was performed using G*Power Statistical software (Germany). Data have been presented as percentages with corresponding 95% confidence intervals. Analysis of all outcomes will be performed on an intention‐to‐treat basis, ensuring that all randomized participants are included in their assigned groups regardless of adherence or withdrawal.

## 4 Results

**FIGURE 1:**
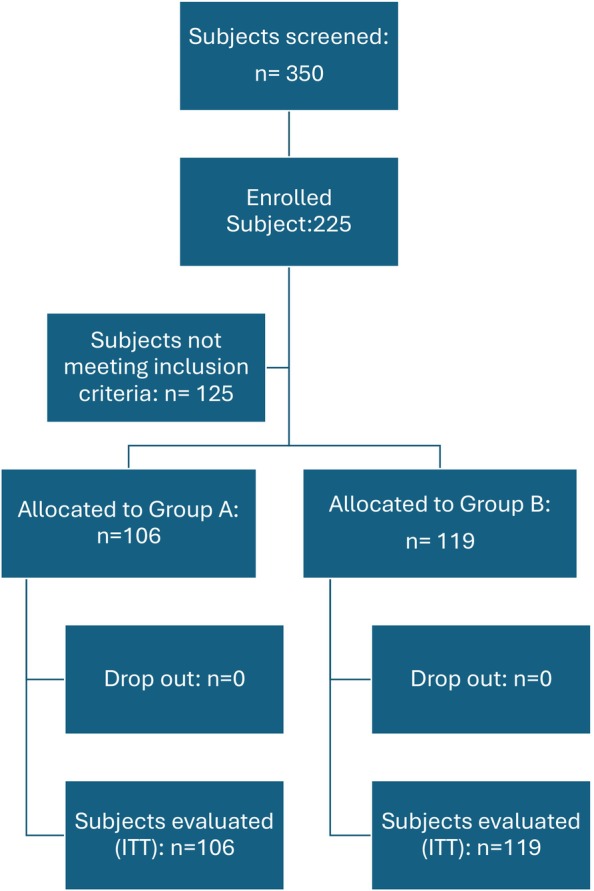
Study flow.

**FIGURE 2:**
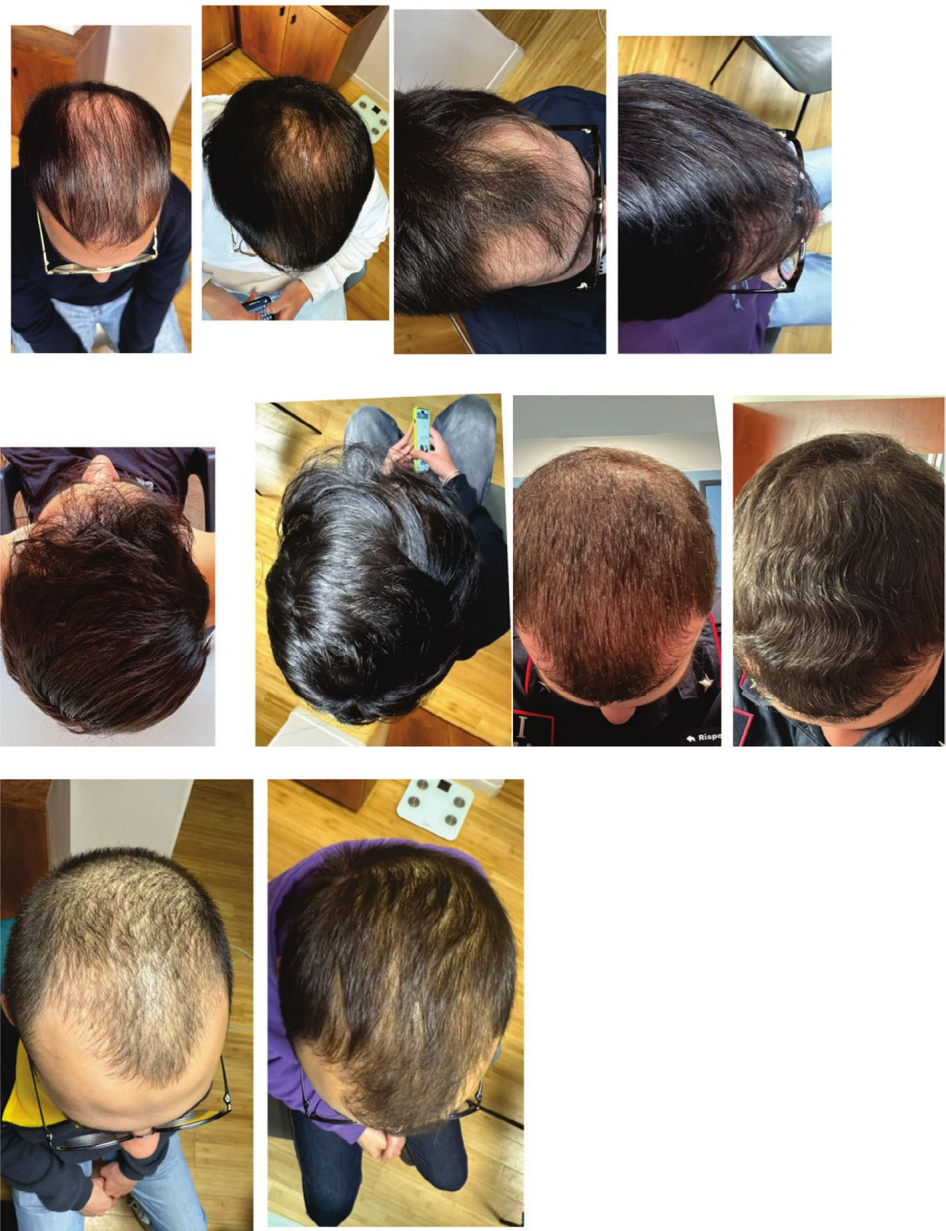
Pictures of five subjects in the Group A at baseline and Month 6.

All enrolled participants completed the 6‐month trial without any dropouts. Figure 1 shows the study flow. At the 6‐month mark, the clinical outcomes were noteworthy: Group A: 36.5% of participants (39 out of 106) (95% CI: from 28% to 48%) achieved a GAS of +3. In Group B, 24% of participants (29 out of 119) (95% CI: from 15% to 31%) achieved the same score. The difference between the two groups was statistically significant (*p* = 0.0428, chi‐square test). Global efficacy evaluations were corroborated by participant feedback, indicating a favorable perception of treatment effectiveness in Group A (46% of subjects reporting great improvement) compared to Group B (30% of subjects reporting great improvement; *p* = 0.0136; chi square test). Regarding the clinical global average score at Month 6, this was 2.0 ± 0.9 in Group A and 1.7 ± 1.0 in Group B (*p* = 0.07; Wilcoxon Test). Figure 2 reports the pictures of five subjects in Group A at baseline and after 6 months of treatment.

### 4.1 Safety Profile

The dietary supplement was well tolerated among all participants, with minimal reported side effects that did not warrant discontinuation of treatment.

## 5 Discussion

The results of the present trial substantiated the hypothesis that the dietary supplement AGA‐P increased the efficacy of standard pharmacological treatments significantly more than pharmacological treatment alone. The addition of natural supplements, such as *S. Repens* and *C. pepo* , can play a crucial role in improving clinical outcomes for individuals with AGA/FAGA [13]. The potential mechanisms through which AGA‐P enhances treatment efficacy could include several mechanisms of action. *S. Repens* is known to inhibit 5‐alpha‐reductase, an enzyme that converts testosterone to dihydrotestosterone (DHT), which contributes to hair loss [14]. *C. Pepo* contains phytosterols and antioxidants that may help in inhibiting DHT formation [15]. L‐Cystine is an amino acid that plays a pivotal role in keratin synthesis, which is vital for hair structure [16]. The antioxidant properties of L‐Cystine help combat oxidative stress, a contributing factor to hair loss and follicle aging [17]. Finally, Vitamin C acts as an antioxidant and supports collagen synthesis, potentially enhancing overall scalp health [18]. Therefore, the combined action of these supplements may synergistically enhance hair growth and reduce hair loss in AGA/FAGA patients compared to standard pharmacological treatments alone. The main results of our study demonstrated that the percentage of patients achieving a score + 3 (much improvement) was significantly higher in the dietary supplementation group in comparison with the drug‐treatment alone group (36.5% vs. 24%). These results were also confirmed by patients subjective evaluation of the clinical efficacy (46% vs. 30%). Some trial limitations should be taken into account in evaluating our results. Even if we have enrolled more than 200 subjects, a larger sample might provide a better understanding of the clinical variation we have documented. A second limit of the study was the trial duration. A 6‐month period may not capture long‐term effects; however, longitudinal studies could build on this data. Not hair counting was performed, but in view of the real‐life nature of this multicenter trial, we decided to evaluate a more feasible clinical efficacy endpoint. Therefore, our study relies on GAS, a subjective visual assessment, rather than objective measures like hair count or density, and this aspect limits comparability with other studies using quantitative endpoints. A relevant percentage of subjects 44% in Group A and 39% in Group B used galenic formulations. This heterogeneity could confound results; however, the use of galenic formulations by Italian dermatologists is quite common, and this aspect is correlated with the real‐life nature of the trial. These formulations generally are made using minoxidil, latanoprost, cetirizine, and/or finasteride using different concentrations. The relevant aspect for the validity of our trial is that the “galenic groups” are comparable in both arms, so this should not be considered a confounding variable. The primary endpoint of our study was not to assess the efficacy of galenic products but to assess if the oral dietary supplementation can increase the efficacy of the main drug treatment chosen by dermatologists in real‐life conditions. Finally, our study was not a double‐blind trial. To increase the internal validity of our results, we adopted the assessor‐blinded approach for the evaluation of the primary endpoint.

## 6 Conclusion

The real‐life randomized trial demonstrated that the oral supplementation of AGA‐P significantly increases the clinical efficacy of pharmacological treatments for mild‐to‐severe AGA/FAGA. This finding highlights the potential of integrating dietary supplements in alopecia treatment strategies, advocating for further research and larger‐scale studies to substantiate these findings.

## Author Contributions

Massimo M. and S.A. designed the research study. Massimo M. wrote the final version of the manuscript. L.A., L.C., A.C., G.C., A.D.S., P.F., G.G., V.G., S.L., L.M., Marco M, F.P., and E.T. performed the research and the study visits.

## Conflicts of Interest

Massimo Milani and Stefano Alfano are employees of Cantabria Labs Difa Cooper, the company selling the AGA‐P product. All other authors have no conflicts of interest.

## Data Availability

The data that support the findings of this study are available on request from the corresponding author. The data are not publicly available due to privacy or ethical restrictions.
